# First Molecular Evidence of Pathogens in Fleas Collected from Dogs in Northern Vietnam

**DOI:** 10.3390/pathogens10091185

**Published:** 2021-09-14

**Authors:** Thom Do, Tawin Inpankaew, Duc Hieu Duong, Khanh Linh Bui

**Affiliations:** 1 Department of Parasitology, Faculty of Veterinary Medicine, Kasetsart University, Bangkok 10900, Thailand; dothanhthom.t@ku.th (T.D.); tawin.i@ku.th (T.I.); 2 Department of Parasitology, Faculty of Veterinary Medicine, Vietnam National University of Agriculture, Hanoi 12406, Vietnam; ddhieu@vnua.edu.vn

**Keywords:** dogs, fleas, Vietnam, flea-borne pathogens

## Abstract

Fleas are considered as hosts for a wide range of pathogens that cause emerging and re-emerging zoonotic diseases worldwide. Data on fleas and flea-borne pathogens (FBPs) in the international literature are limited in Vietnam. This study aimed to investigate the species of fleas and the presence of pathogens of interest in fleas in northern Vietnam using PCR and sequence analysis. Out of 200 dogs enrolled in this study, 20% were infested by the flea species *Ctenocephalides felis felis*. In total, 62 fleas (35 females and 27 males) collected from domestic dogs were molecularly screened for the detection of pathogens. Out of the screened fleas, 39 were positive for *Rickettsia felis* (62.9%), 9 for *Candidatus* Mycoplasma hemobos (14.52%), and 6 for *Mycoplasma wenyonii* (9.68%). This study shows the first molecular detection of the above-mentioned pathogens in fleas collected from the studied areas and the potential risk of infection with examined FBPs in northern Vietnam.

## 1. Introduction

The blood-feeding behavior of fleas (Insecta, Siphonaptera) and their persistence in the environment make them important vectors of an array of pathogens to animals [1,2]. Of particular importance for companion animals are the cat flea, *Ctenocephalides felis felis*, and the dog flea, *Ctenocephalides canis,* which are distributed widely throughout the world. Of those, *C. felis felis* are found much more frequently than *C. canis* on cats and dogs in many temperate and tropical regions, and represent the great majority of fleas in human homes [2]. The prevalence of flea infestations on companion animals was reported as varying from 10.0% to 40.0% in European countries [3,4,5,6], and 14.8% to 19.6% in Asian countries [7]. Infestation rates are highly variable and depend on the location (rural or urban), animal’s lifestyle (outdoor or indoor), sampling season (winter or autumn), and the frequency of effective insecticidal treatments [8].

In addition to the direct effects resulting from blood feeding, *Ctenocephalides* species are also known as vectors of various pathogens, many of which are zoonotic [9]. The current most common disease spread by cat fleas is flea-borne spotted fever, caused by *Rickettsia felis,* which has been reported around the world [2]. *R. felis* has been frequently detected in dogs from Cambodia and China [10,11] and in *C*. *felis* from Taiwan, Laos, Malaysia [12,13], Indonesia, the Philippines, Singapore, and Thailand [7]. Moreover, two species of hemoplasma, *Mycoplasma hemocanis* and *Candidatus* Mycoplasma hematoparvum, have been reported in dogs, which can cause hemolytic anemia in acute infections [14]. The natural route of transmission of these pathogens between dogs has not been determined yet, but the possible role of an arthropod vector is supported by the detection of hemoplasma DNA in fleas collected from dogs [8]. A limited number of studies on flea-borne pathogens (FBPs) are available in Vietnam [7]; therefore, the aim of this study was to perform a molecular detection of bacterial pathogens of interest (*Rickettsia* spp. and *Mycoplasma* spp.) in fleas collected from dogs in northern Vietnam.

## 2. Results

### 2.1. Identification of Fleas and Molecular Detection of Pathogens in Fleas

Of 200 examined dogs, under-1-year-old dogs were abundant at a rate of 59.5%, followed by 1- to-3-year-old dogs (26%), and over-3-year-old dogs (14.5%). Nearly half of the studied dogs were male (42.5%), and most of the dogs were semi-outdoor living (65%). During the sampling, 20% (40 of 200) of the dogs were infested with fleas; however, there were no significant statistical differences between flea infestation and dogs categorized in different groups including age, gender, and living condition (Table 1)**.** All 62 fleas (35 females and 27 males) collected from the domestic dogs were molecularly identified as *Ctenocephalides felis felis*, and the sequences obtained from this study were 99.67–100% identical to previously submitted sequences in Genbank (Accession numbers KX467335 and KP687808). The consensus sequences were submitted to Genbank under the accession numbers MW850560 and MW850561. 

The detection rate of *C. felis felis* in the dogs differed among the studied areas and ranged from 9.6% to 45.8%**.** Of the 62 fleas tested, 39 (62.9%) were positive for *Rickettsia felis* by PCR, 9 (14.52%) for *Ca.* M. hemobos, and and 6 (9.68%) for *M. wenyonii*. The overall detection rate of the selected FBPs in the present study was 87.1% (54/62, 95% CI: 82.1–92.1). For each FBP detected in the fleas, positive amplicons were subjected to sequencing and BLAST analysis. All obtained sequences of *R. felis, Ca.* M. hemobos, and *M. wenyonii* in the current study showed high identity (99.7–100%) with published sequences in Genbank *Rickettsia felis* (Accession numbers AFQ99215 and AFQ99217), *Ca.* M. hemobos (Accession number CP003703) and *M. wenyonii* (Accession number MT241316). Consensus sequences from the present study were submitted to Genbank under the accession numbers MW864274–MW864277 (*R. felis*), MZ063693–MZ063694 (*Ca.* M. hemobos), and MZ043580–MZ043581 (*M. wenyonii*).

Besides single infection, the current study also recorded the occurrence of mixed-pathogen infection in the examined fleas. Particularly, four fleas (6.45%) were positive with both *R. felis* and *Ca.* M. hemobos, while a concurrent infection of *R. felis* and *M. wenyonii* was found in five flea samples (8.06%) (Table 2).

### 2.2. The Phylogenetic Analysis of Rickettsia felis and Hemoplasmas

**Figure 1:**
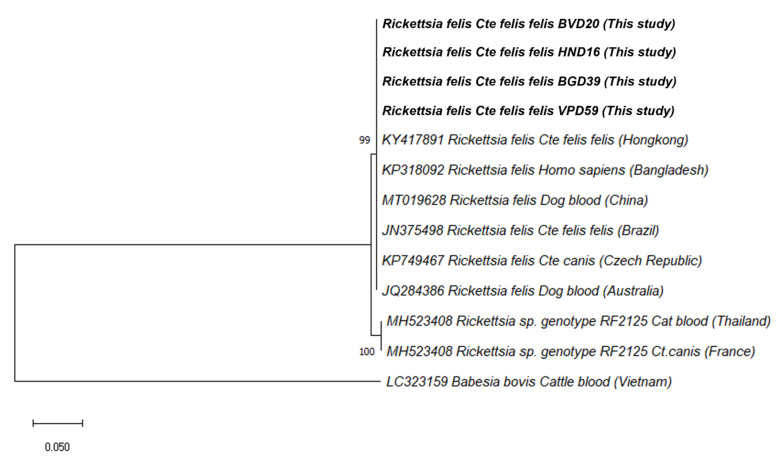
Phylogenetic relationships of *Rickettsia felis* isolated in this study (bolded) to other *Rickettsia felis* samples based on partial sequences of the *gltA* gene.

Phylogenetic analysis of the 595-bp fragment of the *gltA* gene of *R. felis* from fleas in this study with the corresponding available isolates in the database was built based on the maximum likelihood method with the Kimura 2-parameter (Figure 1). All the *R. felis* isolates from this study were found in polytomy with sequences from China (MT019628), Hong Kong (KY417891), Bangladesh (KP318092), Brazil (JN375498), the Czech Republic (KP749467), and Australia (JQ284386) with high bootstrap support (100%) (Supplementary Table S2).

**Figure 2:**
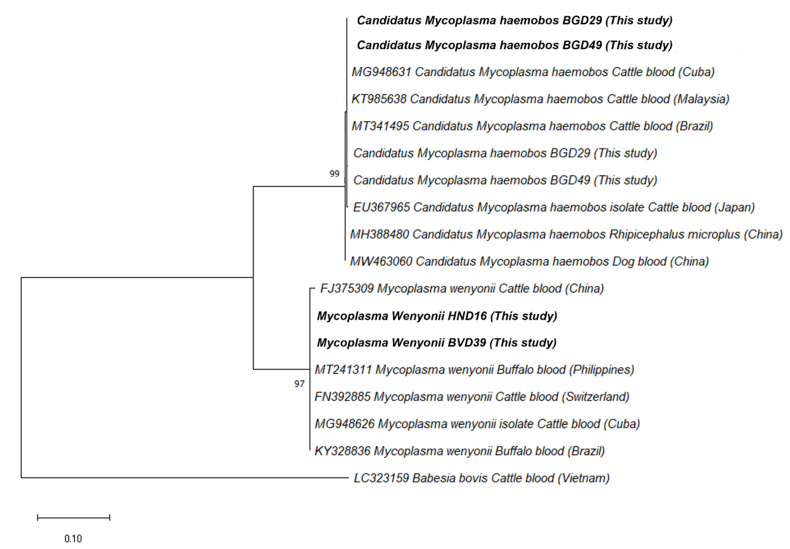
Phylogenetic relationships of hemoplasma isolated in this study (bolded) to other specimens based on partial sequences of the 16S rRNA gene.

In addition, the 431-bp fragment of the 16S rRNA sequences of hemoplasma obtained from this study and another 16S rRNA sequences retrieved from Genbank were used to establish a phylogenetic tree (Figure 2). The sequences of *Ca.* M. hemobos and *M. wenyonii* found in the present study are located in different clades. The isolates showed a high similarity to the strains obtained from cattle blood in Malaysia (KT990216), the Philippines (MT241311), Cuba (MG948626), and Brazil (KY328836) with high bootstrap support (100%) (Supplementary Table S1). 

## 3. Discussion

Fleas are among the most important hematophagous arthropod vectors accountable for the transmission of several pathogens [1,15]. All the fleas collected from dogs in this study were *C. felis felis*. Although *C. felis felis* are cat fleas, they were discovered to be the most common flea species infesting dogs in Vietnam, and have been found more frequently than *C. canis* on dogs in many temperate and tropical regions [2,7]. The occurrence rate of flea infestation (20%; 40/200) found in dogs of this study is quite similar to that of other reported studies in Indonesia and Thailand (16.8–20.8%), but seems higher than those of China, Taiwan, and Singapore (0.9–9.1%) [16]. *C. canis* and *C. orientis* have been detected in investigative studies in Vietnam and several Southeast Asian countries [7,16], but the absence of these species in the current study might be due to the limited sample size and geographical differences in the studied areas. In agreement with other published reports, the present study also showed the absence of statistically significant variations in the overall occurrence rates of flea infestations among dogs of different ages and sex groups [6,17].

The results of this study revealed the occurrence of pathogens in fleas including *Rickettsia felis*, *Ca.* M. hemobos, and *M. wenyonii*, of which, *R. felis* was the most PCR-detected intracellular pathogen (39/62, 62.9%). The phylogenetic tree based on the partial *gltA* gene sequences confirmed that all the *R*. *felis* isolated from fleas in this study assembled together with other reported genotypes isolated from China, Hong Kong, Bangladesh, Brazil, the Czech Republic, and Australia in one cluster (Figure 1). Some of these reports revealed *R. felis* found in dog fleas (*Ctenocephalides canis*) and cat fleas (*Ctenocephalides felis felis*) collected from owned dogs in the Czech Republic and from stray dogs in Hong Kong, respectively. Other reports showed the presence of *R. felis* from dog blood in China and Australia, and one report found it from human blood in Bangladesh. It was found that the occurrence rate of *R. felis* in fleas in this study seemed higher in comparison with other earlier reports elsewhere in Southeast Asia such as Indonesia, the Philippines, Taiwan, and Thailand, which ranged between 9.09% and 33.3% [7]. The difference in the frequency of *R. felis* observed in this study with respect to others could be attributed to the difference in the sampling size, the geographical area, the sampling season, and the target gene employed. In Southeast Asia, rickettsial diseases have been deemed the second-most-common cause of non-malaria febrile illness after Dengue infection [16,18], and *R. felis* is responsible for many cases of febrile illness in Thailand [19] and in Africa [20]. In Vietnam, the spotted fever group (SFG) of rickettsial infections caused by *R. felis* was not well known until 2019, when the first evidence of *R. felis* was found in a case–control study showing that 41 (10.8%) out of 378 febrile adult patients were positive with rickettsia agents, of which 4.9% (2/41) were identified as *R. felis* [21]. The finding of the high occurrence of *R*. *felis* in *C*. *felis felis*, along with the frequency of this flea species in dogs in this study, emphasized the risk of *R*. *felis* transmission in animals and humans in these studied areas. In addition to fleas, ticks, such as *Rhipicephalus sanguineus,* and mosquitoes, such as *Anopheles gambiae*—the major vector of malaria—may also transmit *R. felis* [22]. This point emphasizes the need of epidemiology surveys of this bacterium in dogs and their different vectors in order to find the distribution of this pathogen in Vietnam.

Unexpectedly, of the FBPs detected, *Ca*. M. hemobos and *M. wenyonii*—bovine hemoplasma—were identified in fleas collected from the dogs. *Ca*. M. hemobos and *M. wenyonii* are mainly detected in cattle, causing anemia, anorexia, weight loss, and decreased milk production [23]. The attempt to analyze the phylogeny of hemoplasma sequences from this study showed that there was a high similarity between the hemoplasma strains obtained from fleas and those from cattle blood (Figure 2). The present study found that all of the dogs whose fleas were screened with bovine hemoplasma were actually living on a farm sharing their living environment with other animals such as poultry and cattle. This result might reveal the fact that the cattle population in the studied areas were infected with *Ca*. M. hemobos and *M. wenyonii*, and fleas might have acquired the pathogens in a blood meal taken from an infected host, since the possible transmission routes of bovine hemoplasma are fleas [24], and then had contact with other animals nearby. However, the lack of screening for flea-borne pathogens in dog and cattle blood was a drawback of the present study in order to conclude whether dogs may play a peculiar role as bridging hosts for fleas of different wild animals, domestic animals, and humans [1]. A further experimental study should be conducted to clarify the role of fleas as a vector in hemoplasma transmission and the relationship between hemoplasma or fleas and different hosts. The current study emphasizes the need for large scale epidemiology surveys of this bacterium in dogs and their vectors in order to determine to what extent dogs are important reservoir hosts for vector-borne disease infections in Vietnam.

## 4. Materials and Methods

### 4.1. Study Area and Sample Collection

**Figure 3:**
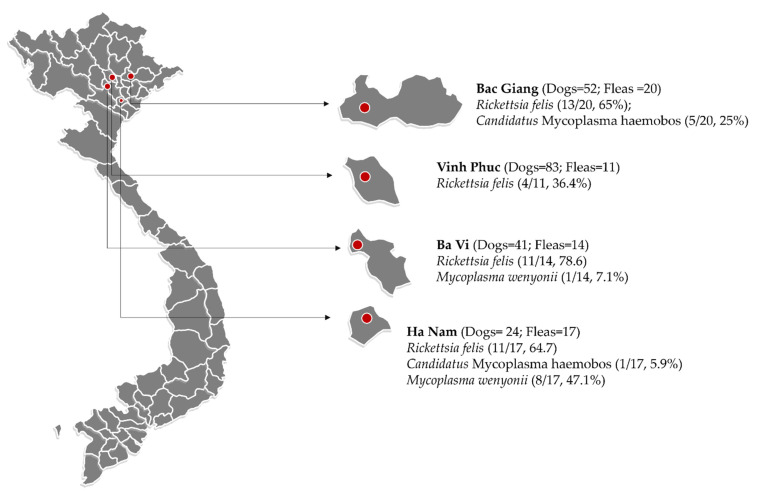
Map of study areas in northern Vietnam where dog flea samples were collected (red circles are representative for sampling sites in each provinces).

Sixty-two fleas were collected from forty privately owned, flea-infested dogs residing in rural areas of several provinces in northern Vietnam (i.e., Ba Vi, Vinh Phuc, Bac Giang, and Ha Nam) from March to May 2018 (Figure 3). In total, 1 to 2 fleas from each dog were collected by combing, and hand-removed and placed in 1.5 mL tubes containing 70% ethanol for later molecular identification. 

### 4.2. DNA Extraction and Molecular Detection

Flea samples were prepared before DNA extraction. Briefly, after removing any residual ethanol by washing the fleas with phosphate-buffer saline, the fleas were ground thoroughly using a sterilized micropestle, and then the tubes were placed in a boiling water bath with Proteinase K for 12 h. Genomic DNA from the fleas was extracted using an E.Z.N.A.^®^ Tissue DNA Kit (Omega Biotek Inc., Norcross, GA, USA) following the manufacturer’s instructions. Subsequently, conventional PCRs were employed to test the DNA samples for the presence of *Ctenocephalides* spp., *Rickettsia* spp., and *Mycoplasma* spp. (Table 3). All DNA amplifications were performed in a 25 µL reaction mixture composed of distilled deionized water, 1 µL of template DNA, 10 pmol of each primer, 10 mM of each deoxynucleotide triphosphate, 2.5 µL of 10× buffer, and 0.13 units of Taq DNA polymerase (BioFact^TM^, Daejeon, South Korea). Amplifications were performed using an Eppendorf MasterCycler Nexus Gradient Thermal Cycler (Eppendorf AG, Hamburg, Germany) under the previously described conditions with some modifications shown in Table 3. Negative controls (distilled deionized water) and positive controls (positive DNA of each pathogen extracted from the blood of infected dogs) were used in each PCR reaction. The PCR products were checked by electrophoresis in 1.5% agarose gel (LE agarose, Thermo Fisher Scientific, Waltham, MA, USA) and TAE (Tris-acetate-EDTA) buffer.

### 4.3. Phylogenetic Analysis 

For sequence analysis, the positive amplicons were snipped from the gel and purified using a QIAquick Gel Extraction Kit (QIAGEN GmbH, Hilden, Germany) according to the manufacturer’s instructions. Subsequently, the purified products were submitted for Sanger DNA sequencing (Macrogen, Seoul, Korea). After obtaining the sequence results, the sequences were compared to published isolates using the Basic Local Alignment Search Tool (BLAST) of the U.S. National Center for Biotechnology Information (https://blast.ncbi.nlm.nih.gov/Blast.cgi, accessed on 20 August 2021), and alignment was achieved using the Finch TV program.

The genetic relationships of the *gltA* gene-based *Rickettsia* and 16S rRNA-based hemoplasma selected in the present study and those from other regions of the world were established by phylogenetic analyses using the MEGA X program, respectively. The maximum likelihood method with the Kimura 2-parameter model was employed to construct the phylogenetic trees [28]. In the representing tree, *Babesia bovis* was used as the outgroup species to root the tree. Bootstrap analysis with 1000 replications was set to estimate the reliability of the branching patterns of the trees. Genbank accession numbers, isolation sources, and countries of origin are presented for each sequence.

## 5. Conclusions

This study provides the first molecular detection of *R*. *felis* and hemoplasma in fleas collected from dogs in northern Vietnam. Our study demonstrates that fleas may play important roles in increasing the risk of mammalian exposure to these pathogenic bacteria in the studied areas. These findings underline the need to perform additional epidemiology surveys in different animal and arthropod species of these zoonotic bacteria to understand and control the complexity of distributions of FBPs and their vectors in Vietnam.

## Figures and Tables

**Table 1:** Flea infestation rate of 200 dogs in northern Vietnam.

Factors	No. Dogs (%) *n* = 200	No. Flea-Infested Dogs (%) *n* = 40	Chi-Square Test
Age (year)			X-squared = 1.31, df = 2, *p*-value = 0.52
<1	119 (59.5)	23 (19.33)	
1–3	52 (26)	9 (17.31)	
>3	29 (14.5)	8 (27.59)	
Gender			X-squared = 0, df = 1, *p*-value = 1
Male	85 (42.5)	17 (20)	
Female	115 (57.5)	23 (20)	
Living condition			X-squared = 0.31, df = 1, *p*-value = 0.6
Outdoor	70 (35)	16 (22.86)	
Semi-outdoor	130 (65)	24 (18.46)	

**Table 2:** The occurrence rate of single- and mixed-pathogen infection in flea samples.

Pathogens	No. Fleas Examined (*n* = 62)	Detection Rate %	95% Confidence Interval
Single infection			
*Rickettsia felis*	39	62.90	55.7–70.1
*Candidatus* Mycoplasma hemobos	9	14.52	9.3–19.7
*Mycoplasma wenyonii*	6	9.68	5.3–14.1
Mixed infection			
*Rickettsia felis* and *Candidatus* Mycoplasma hemobos	4	6.45	2.8–10.1
*Rickettsia felis* and *Mycoplasma wenyonii*	5	8.06	4.01–12.1
Total	54	87.09	82.1–92.1

**Table 3:** Sequences of the primer sets used for flea and flea-borne pathogen detection by PCR in this study.

Pathogen	Oligonucleotide Sequences (5′–3′)	Product Size (bp)	PCR Protocol	Reference
*Flea species* (*Cox 1*)	F: TTATCTGTTTATGTTATATAAGC R: CAGTACCTATGCATATCAATCC	601	94 °C for 3 min initial denaturation, followed by 35 cycles of 94 °C for 30 s, 54.3 °C for 30 s, 72 °C for 30 s, then 72 °C for 5 min for the final elongation	[25]
*Rickettsia felis* (*gltA*)	F1: GCAAGTATTGGTGAGGATGTAATC R1: CTGCGGCACGTGGGTCATAG F2: GCGACATCGAGGATATGACAT R2: GGAATATTCTCAGAACTACCG	654	95 °C for 3 min initial denaturation, followed by 40 cycles of 95 °C for 20 s, 55 °C for 20 s, 72 °C for 20 s, then 72 °C for 5 min for the final elongation	[26]
*Mycoplasma* spp. (16S rRNA)	F: ATACGGCCCATATTCCTACG R: TGCTCCACCACTTGTTCA	595–618	94 °C for 5 min initial denaturation, followed by 40 cycles of 95 °C for 30 s, 60 °C for 30 s, 72 °C for 30 s, then 72 °C for 10 min for the final elongation	[27]

Abbreviations: F: forward, R: reverse.

## Data Availability

The data presented in this study are available on request from the corresponding author.

## Supplementary Materials

The following are available online at https://www.mdpi.com/article/10.3390/pathogens10091185/s1, Table S1: Part of pairwise distance matrix based on nucleotide sequences of *Candidatus* Mycoplasma hemobos and *Mycoplasma wenyonii* strains. Table S2: Part of pairwise distance matrix based on nucleotide sequences of *Rickettsia* sp. strains.

## References

[1] Dobler G., Pfeffer M. (2011). Fleas as parasites of the family Canidae. Parasites Vectors.

[2] Bitam I., Dittmar K., Parola P., Whiting M.F., Raoult D. (2010). Fleas and flea-borne diseases. Int. J. Infect. Dis..

[3] Beck W., Boch K., Mackensen H., Wiegand B., Pfister K. (2006). Qualitative and quantitative observations on the flea population dynamics of dogs and cats in several areas of Germany. Veter-Parasitol..

[4] Bond R., Riddle A., Mottram L., Beugnet F., Stevenson R. (2007). Survey of flea infestation in dogs and cats in the United Kingdom during 2005. Vet. Rec..

[5] Beugnet F., Franc M. (2010). Results of a European multicentric field efficacy study of fipronil-(S) methoprene combination on flea infestation of dogs and cats during 2009 summer. Parasite.

[6] Farkas R., Gyurkovszky M., Solymosi N., Beugnet F. (2009). Prevalence of flea infestation in dogs and cats in Hungary combined with a survey of owner awareness. Med. Vet. Èntomol..

[7] Nguyen V.-L., Colella V., Greco G., Fang F., Nurcahyo W., Hadi U.K., Venturina V., Tong K.B.Y., Tsai Y.-L., Taweethavonsawat P. (2020). Molecular detection of pathogens in ticks and fleas collected from companion dogs and cats in East and Southeast Asia. Parasites Vectors.

[8] Abdullah S., Helps C., Tasker S., Newbury H., Wall R. (2019). Pathogens in fleas collected from cats and dogs: Distribution and prevalence in the UK. Parasites Vectors.

[9] Beugnet F., Marie J.L. (2009). Emerging arthropod-borne diseases of companion animals in Europe. Vet. Parasitol..

[10] Inpankaew T., Hii S.F., Chimnoi W., Traub R.J. (2016). Canine vector-borne pathogens in semi-domesticated dogs residing in northern Cambodia. Parasites Vectors.

[11] Zhang J., Lu G., Kelly P., Zhang Z., Wei L., Yu D., Kayizha S., Wang C. (2014). First report of *Rickettsia felis* in China. BMC Infect. Dis..

[12] Tsai K.-H., Huang C.-G., Fang C.-T., Shu P.-Y., Huang J.-H., Wu W.-J. (2011). Prevalence of *Rickettsia felis* and the First Identification of *Bartonella henselae* Fizz/CAL-1 in Cat Fleas (*Siphonaptera: Pulicidae*) From Taiwan. J. Med. Èntomol..

[13] Kernif T., Socolovschi C., Wells K., Lakim M.B., Inthalad S., Slesak G., Boudebouch N., Beaucournu J.-C., Newton P.N., Raoult D. (2012). Bartonella and Rickettsia in arthropods from the Lao PDR and from Borneo, Malaysia. Comp. Immunol. Microbiol. Infect. Dis..

[14] Compton S.M., Maggi R.G., Breitschwerdt E.B. (2012). *Candidatus Mycoplasma haematoparvum* and *Mycoplasma haemocanis* infections in dogs from the United States. Comp. Immunol. Microbiol. Infect. Dis..

[15] Reif K.E., Macaluso K.R. (2009). Ecology of *Rickettsia felis*: A Review. J. Med. Èntomol..

[16] Robinson M.T., Satjanadumrong J., Hughes T., Stenos J., Blacksell S.D. (2019). Diagnosis of spotted fever group Rickettsia infections: The Asian perspective. Epidemiol. Infect..

[17] Gracia M.J., Calvete C., Estrada R., Castillo J.A., Peribáñez M.A., Lucientes J. (2008). Fleas parasitizing domestic dogs in Spain. Vet. Parasitol..

[18] Acestor N., Cooksey R., Newton P.N., Menard D., Guerin P.J., Nakagawa J., Christophel E., Gonzalez I.J., Bell D. (2012). Mapping the aetiology of non-malarial febrile illness in Southeast Asia through a systematic review—terra incognita impairing treatment policies. PLoS ONE.

[19] Edouard S., Bhengsri S., Dowell S.F., Watt G., Parola P., Raoult D. (2014). Two Human Cases of Rickettsia felis Infection, Thailand. Emerg. Infect. Dis..

[20] Brown L.D., Macaluso K.R. (2016). Rickettsia felis, an emerging flea-borne rickettsiosis. Curr. Trop. Med. Rep..

[21] Le-Viet N., Le V.-N., Chung H., Phan D.-T., Phan Q.-D., Cao T.-V., Abat C., Raoult D., Parola P. (2019). Prospective case-control analysis of the aetiologies of acute undifferentiated fever in Vietnam. Emerg. Microbes Infect..

[22] Dieme C., Bechah Y., Socolovschi C., Audoly G., Berenger J.-M., Faye O., Raoult D., Parola P. (2015). Transmission potential of Rickettsia felis infection by Anopheles gambiae mosquitoes. Proc. Natl. Acad. Sci. USA.

[23] Baggenstos R., Wenzinger B., Meli M.L., Hofmann-Lehmann R., Knubben-Schweizer G. (2012). Haemoplasma infection in a dairy cow. Tierärztliche Prax. Ausg. G Großtiere Nutztiere.

[24] Hornok S., Meli M.L., Perreten A., Farkas R., Willi B., Beugnet F., Lutz H., Hofmann-Lehmann R. (2010). Molecular investigation of hard ticks (Acari: Ixodidae) and fleas (*Siphonaptera: Pulicidae*) as potential vectors of rickettsial and mycoplasmal agents. Vet. Microbiol..

[25] Lawrence A.L., Hii S.-F., Jirsová D., Panáková L., Ionică A.M., Gilchrist K., Modrý D., Mihalca A.D., Webb C.E., Traub R.J. (2015). Integrated morphological and molecular identification of cat fleas (*Ctenocephalides felis*) and dog fleas (*Ctenocephalides canis*) vectoring *Rickettsia felis* in central Europe. Vet. Parasitol..

[26] Hii S.-F., Kopp S.R., Thompson M.F., O’Leary C.A., Rees R.L., Traub R.J. (2011). Molecular evidence of *Rickettsia felis* infection in dogs from northern territory, Australia. Parasites Vectors.

[27] Criado-Fornelio A., Martinez-Marcos A., Buling-Saraña A., Barba-Carretero J. (2003). Presence of *Mycoplasma haemofelis*, *Mycoplasma haemominutum* and piroplasmids in cats from southern Europe: A molecular study. Vet. Microbiol..

[28] Nishimaki T., Sato K. (2019). An Extension of the Kimura Two-Parameter Model to the Natural Evolutionary Process. J. Mol. Evol..

