# Supplementary material for: First Molecular Evidence of Pathogens in Fleas Collected from Dogs in Northern Vietnam
Source: Pathogens. 2021 Sep 14;10(9):1185. doi: 10.3390/pathogens10091185 (PMC8472576; doi:10.3390/pathogens10091185)
Supplement: Supplementary file 1 [file pathogens-10-01185-s001.zip › pathogens-1331485-supplementary.pdf]

Table S1. Part of pairwise distance matrix based on nucleotide sequences of *Candidatus* Mycoplasma haemobos and *Mycoplasma wenyonii* strains.

**No.**		**1**	**2**	**3**	**4**	**5**	**6**	**7**	**8**	**9**	**10**	**11**	**12**	**13**	**14**	**15**	**16**	**17**
**1**	*Ca*. M. haemobos BGD29 (Thisstudy)																	
**2**	*Ca*. M. haemobos BGD49 (This study)	0.000																
**3**	MT341495 *Ca*. M. haemobos Cattle blood (Brazil)	0.002	0.002															
**4**	KT985638 *Ca*. M. haemobos Cattle blood (Malaysia)	0.002	0.002	0.000														
**5**	MG948631 *Ca*. M. haemobos Cattle blood (Cuba)	0.002	0.002	0.000	0.000													
**6**	KJ883514 *Ca*. M. haemobos Dog blood (Taiwan)	0.002	0.002	0.000	0.000	0.000												
**7**	EF616468 *Ca*. M. haemobos Cattle Blood (Switzerland)	0.002	0.002	0.000	0.000	0.000	0.000											
**8**	MH388480 *Ca*. M. haemobos *Rhipicephalus microplus* (China)	0.004	0.004	0.002	0.002	0.002	0.002	0.002										
**9**	MW463060 Ca. M. haemobos Dog blood (China)	0.004	0.004	0.002	0.002	0.002	0.002	0.002	0.000									
**10**	EU367965 Ca. M. haemobos Cattle blood (Japan)	0.004	0.004	0.002	0.002	0.002	0.002	0.002	0.004	0.004								
**11**	*M. Wenyonii* HND16 (This study)	1.399	1.399	1.397	1.397	1.397	1.397	1.397	1.394	1.394	1.399							
**12**	*M. Wenyonii* BVD39 (This study)	1.399	1.399	1.397	1.397	1.397	1.397	1.397	1.394	1.394	1.399	0.004						
**13**	MT241311 *M. wenyonii* Buffalo blood (Philippines)	1.373	1.373	1.370	1.370	1.370	1.370	1.370	1.368	1.368	1.373	0.004	0.004					
**14**	FN392885 *M. wenyonii* Cattle blood (Switzerland)	1.339	1.339	1.336	1.336	1.336	1.336	1.336	1.334	1.334	1.339	0.000	0.004	0.004				
**15**	FJ375309 *M. wenyonii* Cattle blood (China)	1.505	1.505	1.503	1.503	1.503	1.503	1.503	1.501	1.501	1.505	0.015	0.015	0.010	0.014			
**16**	MG948626 *M. wenyonii* Cattle blood (Cuba)	1.373	1.373	1.370	1.370	1.370	1.370	1.370	1.368	1.368	1.373	0.004	0.004	0.000	0.004	0.010		
**17**	KY328836 *M. wenyonii* Buffalo blood (Brazil)	1.512	1.512	1.510	1.510	1.510	1.510	1.510	1.507	1.507	1.512	0.004	0.004	0.000	0.004	0.011	0.000	

Pairwise distances calculations were accomplished using MEGA X software.

Table S2. Part of pairwise distance matrix based on nucleotide sequences of *Rickettsia* sp. strains.

**No.**		**1**	**2**	**3**	**4**	**5**	**6**	**7**	**8**	**9**	**10**	**11**
**1**	*R. felis Cte felis felis* BVD20 (This study)											
**2**	*R. felis Cte felis felis* HND16 (This study)	0.000										
**3**	*R. felis Cte felis felis* BGD39 (This study)	0.000	0.000									
**4**	*R. felis Cte felis felis* VPD59 (This study)	0.000	0.000	0.000								
**5**	KY417891 *R. felis Cte felis felis* (Hongkong)	0.000	0.000	0.000	0.000							
**6**	KP318092 *R. felis* Homo sapiens (Bangladesh)	0.000	0.000	0.000	0.000	0.000						
**7**	MT019628 *R. felis* Dog blood (China)	0.000	0.000	0.000	0.000	0.000	0.000					
**8**	JN375498 *R. felis Cte felis felis* (Brazil)	0.000	0.000	0.000	0.000	0.000	0.000	0.000				
**9**	KP749467 *R. felis Cte canis* (Czech Republic)	0.000	0.000	0.000	0.000	0.000	0.000	0.000	0.000			
**10**	JQ284386 *R. felis* Dog blood (Australia)	0.000	0.000	0.000	0.000	0.000	0.000	0.000	0.000	0.000		
**11**	MH523408 *Rickettsia* sp. genotype RF2125 Cat blood (Thailand)	0.015	0.015	0.015	0.015	0.015	0.016	0.015	0.015	0.015	0.015	
**12**	MH523408 *Rickettsia* sp. genotype RF2125 *Cte canis* (France)	0.015	0.015	0.015	0.015	0.015	0.016	0.015	0.015	0.015	0.015	0.000

Pairwise distances calculations were accomplished using MEGA X software.
